# Impact of Amending the Acetylcysteine Marketing Authorisation on Treatment of Paracetamol Overdose

**DOI:** 10.1155/2013/494357

**Published:** 2013-07-16

**Authors:** G. Thompson, S. B. Fatima, N. Shah, G. Kitching, W. S. Waring

**Affiliations:** ^1^Acute Medical Unit, York Teaching Hospital NHS Foundation Trust, Wigginton Road, York YO31 8HE, UK; ^2^Emergency Department, York Teaching Hospital NHS Foundation Trust, UK

## Abstract

In September 2012, the Medicines and Healthcare products Regulatory Agency (MHRA) substantially amended the Marketing Authorisation for acetylcysteine following an extensive review. The present study examined the impact of this license change on patterns of acetylcysteine use in patients presenting to hospital after paracetamol (acetaminophen) overdose. Between September 2011 and April 2013, 785 consecutive patients presented to York Hospital due to paracetamol overdose, and a before-after analysis was used to compare outcomes. There were 483 patients before and 302 patients after the license amendment, and age, gender, acute or staggered overdose pattern, and dose were similar in both groups. In the patients with paracetamol concentrations between the “100-line” and “200-line,” a significantly higher proportion received acetylcysteine treatment (51% before versus 98% after, *P* = 0.0029), as expected. A modest increase was also observed in relation to late or staggered overdose or cases where the time of ingestion was uncertain (53% versus 74%, *P* = 0.0430). The median duration of hospital stay increased across the entire study population, from 15 to 24 hours (*P* = 0.0159) due to the increased proportion of patients requiring acetylcysteine treatment. The findings indicate that the MHRA amendment is a financially costly intervention, and further studies are needed to examine clinical outcomes so that its cost effectiveness might be addressed.

## 1. Introduction

Acetylcysteine is well established as a safe and effective antidote for paracetamol poisoning, although there is uncertainty regarding the indications for treatment and most effective administration protocol [1–3]. A treatment nomogram was originally devised by Prescott to allow identification of patients at significant risk of acute liver injury, the so-called “200-line” plotted between 200 mg/L (1320 *μ*mol/L) at 4 hours and 30 mg/L (200 *μ*mol/L) at 15 hours [4]. As a modification, the “100-line” was plotted 50% lower so that treatment was indicated by lower paracetamol concentrations in patients with individual risk factors for paracetamol toxicity, such as malnourishment, chronic excess ethanol intake, or prior use of enzyme-inducing drugs [5–7]. These have long been established in clinical practice in the United Kingdom as “standard” and “high-risk” nomograms to indicate the need for acetylcysteine after acute paracetamol overdose. Other protocols have been adopted elsewhere, for example, the Rumack nomogram or “150-line” is used in the United States for all patients irrespective of individual risk factors and is plotted 25% lower than the standard Prescott nomogram and extrapolated to 24 hours [8]. The rationale for treating only patients at increased risk of toxicity is based, at least in part, upon the high rate of occurrence of adverse effects of acetylcysteine particularly amongst patients with comparatively low paracetamol concentrations [9, 10]. A notable exception to this practice is the routine treatment of all paracetamol overdose patients in Denmark, irrespective of paracetamol concentration or risk factors [9, 11].

Despite widespread acceptance of the nomogram method, there are important limitations to its application in clinical practice. These include the following. (1) it is valid only for estimating paracetamol exposure up to 15 hours after single time-point ingestion and cannot be applied where there has been repeated or staggered ingestion over >1 hour. (2) the clinical decision to apply the “200-line” or “100-line” in an individual patient may be difficult due to a lack of objective assessment of risk factors such as malnutrition or chronic alcohol excess [5–7, 12, 13]. (3) the nomogram fails to identify all patients that may develop toxicity and, for example, around 1 in 4400 patients develops acute liver failure despite paracetamol concentrations below the “100-line” [14]. The Medicines and Healthcare products Regulatory Agency (MHRA) undertook a major review of acetylcysteine between 2011 and 2012 and substantially amended its Marketing Authorisation with effect from 3rd September 2012 onward. Amendments included (1) abandoning assessment of individual patient risk factors, (2) application of the “100-line” to all cases so that the “200-line” is obsolete, and (3) treating all patients after staggered overdose or where the time of ingestion is unclear, irrespective of the paracetamol concentrations [15].

The regulatory amendment was based solely on a risk-benefit basis, and resource implications were beyond the remit of the MHRA review. Few clinical data were available to inform the likely number of additional patients that might require treatment. Therefore, the present study sought to examine the impact of the effect of the Marketing Authorisation amendment on patterns of acetylcysteine administration and hospitalisation after single time-point and staggered paracetamol overdose.

## 2. Methods

### 2.1. Study Design

York Hospital serves a catchment population of around 350 thousand people, and receives approximately 70 thousand Emergency Department attendances per year. Adults that present after paracetamol overdose are assessed in a dedicated observation area and may be discharged home after completion of medical and psychiatric assessment or may be admitted to the Acute Medical Unit if ongoing medical treatment is required or the patient is too drowsy or intoxicated to allow detailed psychiatric assessment [16]. The study population consisted of consecutive patients aged ≥16 years that presented to the Emergency Department due to paracetamol overdose between September 2011 and April 2013 inclusive. Data collected were age, gender, weight, date and time of ingestion, paracetamol dose, serum paracetamol concentration, acetylcysteine administration, and duration of hospital episode. 

Paracetamol concentrations are determined using an enzymatic hydrolysis method (Olympus Diagnostics, Southall, United Kingdom) and performed using an AU2700 automated analyser (Beckman Coulter, High Wycombe, United Kingdom). The test principle is based on paracetamol hydrolysis by *aryl acylamidase* to yield p-aminophenol and acetic acid; p-aminophenol further reacts with o-cresol and ammoniacal copper sulphate to form indophenol. The quantity of indophenol is determined using spectrophotometry at a wavelength of 600 nm and is directly proportional to the amount of paracetamol in the sample. The assay is specific for paracetamol and is not subject to interference by its major metabolites [17]. 

### 2.2. Data Analysis

A before-after analysis was used to compare patterns of paracetamol overdose and acetylcysteine administration around the Marketing Authorisation amendment on 3rd September 2012. Paracetamol dose was expressed in grams and mg/kg body weight. If patients presented <15 hours after acute single-time-point ingestion, paracetamol concentrations were considered as above or below the “100-line” and “200-line” nomograms. Data are presented as median and interquartile range and proportions, and between-group comparisons made using Mann Whitney tests and two-tailed Yates corrected Chi-square proportional tests (MedCalc statistical software v.12.5.0.0, Mariakerke, B-8400 Ostend, Belgium). *P* values < 0.05 were accepted as statistically significant in all cases.

## 3. Results

There were 785 patients, including 483 that presented before 3rd September 2012 and 302 that presented after. The age, gender, pattern of paracetamol overdose, quantity ingested, and measured drug concentrations were similar in both groups (Table 1). Paracetamol concentrations were not measured in 61 patients: in 58 cases the paracetamol dose was considered nontoxic (<8 grams) so that drug concentrations were considered unnecessary, and in 3 cases the patient refused blood samples despite being clinically indicated and acetylcysteine was administered without a paracetamol concentration. 

In patients with paracetamol concentrations between the “100-line” and “200-line,” a higher proportion received acetylcysteine after the Marketing Authorisation change (51% before versus 98% after, *P* = 0.0029), as expected; one patient failed to receive acetylcysteine after the license change because a paracetamol concentration of 96 mg/L at 4.3 hours was incorrectly interpreted as below the “100-line”. There was also an increase in the proportion of patients treated if they presented after staggered ingestion >1 hour (54% before versus 80% after, *P* = 0.0498), whereas treatment of other overdose patterns was broadly similar before and after the licence change (Table 2). 

Patients were considered in one of three treatment groups: (1) those that ingested a nontoxic dose (<8 grams) and did not require paracetamol concentration to be checked, (2) those that did not require acetylcysteine treatment, and (3) those that received acetylcysteine treatment. The duration of hospital stay in each treatment group did not change as a result of the Marketing Authorisation update (Table 3). However, across the study population the median duration of hospital stay increased after the license amendment from 15 to 24 hours (*P* = 0.0159) due to an increased number of patients that required acetylcysteine treatment (Table 3). The overall duration of hospital stay was 15696 hours before September 2012 and 11358 hours after, representing an additional 5.1 hours (95% confidence interval 4.3–6.0 hours) for every patient that presented to hospital (*P* < 0.0001). 

## 4. Discussion

These data show that the MHRA amendment to the acetylcysteine Marketing Authorisation led to a significant change in clinical practice after September 2012. As expected, there was a substantial increase in the number of patients receiving treatment if paracetamol concentrations were between the “200-line” and “100-line,” indicating a high level of awareness and implementation of the new recommendations in respect of acute single time-point overdose. The legislative changes in relation to late or staggered overdose or where the time of ingestion was uncertain also led to an increase in the proportion of patients treated (53% to 74%). This indicates a poorer level of awareness and implementation of the updated guidance in relation to staggered overdose and delayed presentations, which are that patterns of paracetamol overdose with the worst outcome. This might be explained by poor communication between the regulatory authorities and prescribers, as previously been suggested [18]. Clinicians would be expected to make reference to the treatment nomogram in management of acute, single time-point overdose, and consultation with an up-to-date resource such as TOXBASE or the British National Formulary might have alerted to the MHRA amendments. In contrast, clinicians may be less likely to access additional resources when faced with a late or staggered overdose. Fewer patients presented late or after a staggered ingestion than after acute overdose, so that experiential learning might take longer to be implemented into clinical practice. 

The MHRA update was associated with an increased hospital utilisation, as expected. The magnitude of that change equated to an additional 5.1 hours for every patient presenting to hospital after paracetamol overdose, which correspond to an additional 102 bed days per year and costs of *£*35–50,000 at York Hospital alone. If the same findings were extrapolated to the United Kingdom, then this would represent an additional cost of around *£*7.0–8.5 million annually. Indeed, the eventual cost is likely to be even greater once the Marketing Authorisation amendments concerning late and staggered overdoses are fully implemented. These data indicate that the recent MHRA amendment will have a high cost implication. The amendment was based primarily upon a risk-benefit analysis that primarily sought to lessen the occurrence of paracetamol-induced liver failure. Clinical experience indicates that acute liver injury is rare in patients that do not meet the criteria for acetylcysteine based upon the “150-line” after acute paracetamol overdose, and there have been calls for the United Kingdom to adopt this approach as used in the United States, Australia, and New Zealand [19, 20]. In order to examine the impact of the MHRA update and its cost effectiveness, multicentre studies will need to recruit very large patient numbers to examine the occurrence of paracetamol-induced liver failure. There are challenges to applying conventional research methods to the study of paracetamol poisoning, but recent studies demonstrate the feasibility of clinical research in this patient group and are encouraging [3, 21].

A limitation is that the findings are based on data from one hospital and might not be generalised to other institutions. Notwithstanding, paracetamol accounts for around 40% of all overdose presentations at York Hospital, which is broadly similar to that at other hospitals in the United Kingdom [22]. A further potential limitation is that the study period extended to only 8 months after the MHRA update, whereas the legislative changes might perhaps take longer than this to become fully implemented into clinical practice [23, 24]. 

## 5. Conclusions

The findings indicate that the update to the Marketing Authorisation for acetylcysteine has led to a significant change in clinical practice. There has been a significant increase in antidote administration after paracetamol overdose, resulting in a significantly prolonged hospital stay overall. This is a financially costly intervention, and further studies are needed to examine clinical outcomes so that the cost-effectiveness of the recent MHRA update can be addressed. 

## Figures and Tables

**Table 1:** Clinical characteristics, laboratory findings, and overdose patterns presented as median (interquartile range), and proportions.

Characteristic	Sept. 2011–Aug. 2012	Sept. 2012–Apr. 2013
Number	483	302
Females	311 (64.4%)	193 (63.6%)
Age (years)	24 (18–44)	24 (17–43)
Weight (kg)	69 (60–82)	70 (60–84)
Paracetamol dose (grams)	10.0 (6.0–16.0)	9.5 (5.0–16.0)
Paracetamol dose (mg/kg)	176 (123–259)	174 (104–287)
Interval overdose to level (hours)	4.4 (4.0–6.0)	4.7 (4.0–6.1)
Paracetamol level (mg/L)	61 (33–98)	59 (32–104)
Equivalent 4-hour level (mg/L)	60 (27–122)	59 (18–111)
Overdose pattern		
Single time-point ingestion, <15 hours		
Above “200-line”	22 (4.6%)	12 (4.0%)
Between “200-line” and “100-line”	76 (15.7%)	45 (14.9%)
Below “100-line”	208 (43.1%)	135 (44.7%)
Nomogram not applicable		
Staggered ingestion (>1 hour)	100 (20.7%)	59 (19.5%)
Late presentation (>15 hours)	23 (4.8%)	13 (4.3%)
Time of ingestion uncertain	18 (3.7%)	13 (4.3%)
Level not checked	36 (7.5%)	25 (8.3%)

**Table 2:** Acetylcysteine administration according to pattern of paracetamol overdose, presented as proportions.

Overdose pattern	Sept. 2011–Aug. 2012	Sept. 2012–Apr. 2013
Single time-point ingestion, <15 hours		
Above “200-line”	22/22 (100%)	12/12 (100%)
Between “200-line” and “100-line”	39/76 (51%)	44/45 (98%)^†††^
Below “100-line”	31/208 (15%)	22/135 (16%)
Nomogram not applicable		
Staggered ingestion (>1 hour)	54/100 (54%)	47/59 (80%)^†^
Late presentation (>15 hours)	12/23 (52%)	9/13 (69%)
Time of ingestion uncertain	8/18 (44%)	7/13 (54%)
Level not checked	2/36 (6%)	1/25 (4%)

Total	168/483 (34.8%)	142/302 (47.0%)^††^

^†^ *P* = 0.0498, ^††^ *P* = 0.0079, ^†††^ *P* = 0.0029 versus earlier time period by Chi-square proportional tests.

**Table 3:** Duration of hospital episode from Emergency Department attendance to discharge, presented in hours as median (interquartile range).

Clinical management	Sept. 2011–Aug. 2012	Sept. 2012–Apr. 2013
Paracetamol level not checked and antidote not administered	*n* = 34 (7.0%) 3.0 (2.0–3.5)	*n* = 24 (7.9%) 2.4 (1.8–3.6)
Paracetamol level checked but antidote not administered	*n* = 281 (58.2%) 7.1 (4.0–20.8)	*n* = 136 (45.0%) 8.6 (4.2–22.3)
Acetylcysteine administered	*n* = 168 (34.8%) 42.4 (28.8–63.6)	*n* = 142 (47.0%) 43.1 (29.7–67.0)

Total	*n* = 483 15.4 (4.6–40.1)	*n* = 302 23.8 (5.6–44.9)^†^

^†^ *P* = 0.0159 versus earlier time period by Mann Whitney test.
